# Adherence to the Mediterranean Diet and Bone Fracture Risk in Middle-Aged Women: A Case Control Study

**DOI:** 10.3390/nu11102508

**Published:** 2019-10-18

**Authors:** Anna Palomeras-Vilches, Eva Viñals-Mayolas, Concepció Bou-Mias, MªÀngels Jordà-Castro, MªÀngels Agüero-Martínez, Montserrat Busquets-Barceló, Georgina Pujol-Busquets, Carme Carrion, Marina Bosque-Prous, Lluís Serra-Majem, Anna Bach-Faig

**Affiliations:** 1 Medicina Familiar i Comunitària (MFiC), Institut Català de la Salut, EAP Santa Clara, 17001 Girona, Spain; apalomeras.girona.ics@gencat.cat (A.P.-V.); conxabou.girona.ics@gencat.cat (C.B.-M.); 2 DUI, Institut Català de la Salut, 17001 Girona, Spain; evinals.girona.ics@gencat.cat (E.V.-M.); mbusquetsb.girona.ics@gencat.cat (M.B.-B.); 3 Faculty of Health Sciences, Universitat Oberta de Catalunya (Open University of Catalonia, UOC), 08018 Barcelona, Spain or pjlgeo001@myuct.ac.za (G.P.-B.); mbosquep@uoc.edu (M.B.-P.); 4 Division of Exercise Science and Sports Medicine, Department of Human Biology, Faculty of Health Sciences, University of Cape Town, 7725 Cape Town, South Africa; 5 UOC eHealth Center (eHC), Universitat Oberta de Catalunya (UOC), 08018 Barcelona, Spain; mcarrionr@uoc.edu; 6 eHealth Lab Research Group, Faculty of Health Sciences, Universitat Oberta de Catalunya (UOC), 08018 Barcelona, Spain; 7 CIBER de Fisiopatología de la Obesidad y la Nutrición (CIBEROBN), Instituto de Salud Carlos III, 28029 Madrid, Spain; lserra@dcc.ulpgc.es; 8 Research Institute of Biomedical and Health Sciences, University of Las Palmas de Gran Canaria, 35001 Las Palmas de Gran Canaria, Spain; 9 FoodLab Research Group (2017SGR 83), Faculty of Health Sciences, Universitat Oberta de Catalunya (Open University of Catalonia, UOC), 08018 Barcelona, Spain; 10 Food and Nutrition Area, Barcelona Official College of Pharmacists, 08009 Barcelona, Spain

**Keywords:** Mediterranean diet, bone fracture risk, osteoporosis, FRAX, dietary pattern, lifestyle

## Abstract

The prevention of bone mass loss and related complications associated with osteoporosis is a significant public health issue. The Mediterranean diet (MD) is favorably associated with bone health, a potentially modifiable risk factor. The objective of this research was to determine MD adherence in a sample of women with and without osteoporosis. In this observational case-control study of 139 women (64 women with and 75 without osteoporosis) conducted in a primary-care health center in Girona (Spain), MD adherence, lifestyle, physical exercise, tobacco and alcohol consumption, pathological antecedents, and FRAX index scores were analyzed. Logistic multilinear regression modeling to explore the relationship between the MD and bone fracture risk indicated that better MD adherence was associated with a lower bone risk fracture. Non-pharmacological preventive strategies to reduce bone fracture risk were also reviewed to explore the role of lifestyle and diet in bone mass maintenance and bone fracture prevention.

## 1. Introduction

The Mediterranean diet (MD) has been recognized as a model of healthy eating because of its contribution to a healthy lifestyle and a better quality of life. The MD is much more than a dietary pattern. The word diet comes from the Greek *diaita* and means ‘way of life’ and the MD holds true to this meaning as MD covers not only what to eat but how to eat and live (active lifestyle, prioritizing culinary activities, etc.) [1,2]. And it should be preserved in the Mediterranean basin where, in the past decades, dietary trends are departing from traditional dietary patterns [3]. The principles underlying the MD—widely documented elsewhere [1]—can be summarized as follows: high consumption of fruits, vegetables, cereals (especially whole seeds), legumes, and nuts; relatively high fat consumption (up to 40% of total energy intake), mainly monounsaturated fatty acids (MUFAs) from extra-virgin olive oil, the main fat used for seasoning and cooking; moderate to high consumption of fish; moderate dairy product consumption, usually in the form of yogurt and cheese; low red meat and meat product consumption; moderate alcohol consumption, mainly in the form of red wine during meals; low consumption of simple sugars (pastries, soft drinks, etc.); and high consumption of herbs and spices—an important source of micronutrients, including calcium (Ca)—used to flavor dishes [1,2]. This traditional diet, however, has been affected by the onslaught of dietary trends of recent decades [1,2,3].

Osteoporosis (OP) is among the diseases with the highest incidence in advanced age, i.e., coronary heart diseases, cancer, respiratory system diseases, depression, and neurodegenerative diseases such as Alzheimer’s disease [4]. Associated with OP, especially prevalent among older women, is bone fracture risk. This growing global health problem [5] suggests that the prevention of OP and its complications and the maintenance of bone mass are public health concerns.

OP is a systemic disease characterized by a low bone mass index and destruction of the microarchitecture of bone mass—i.e., bone mineral density (BMD)—posing a major risk of fractures. There are no symptoms indicating a reduction in bone mass, and fractures are the only clinical consequences of OP; therefore, despite being the most common bone disease, the incidence is difficult to establish [5,6,7], and the prevalence is often based on estimates using indirect indicators such as fractures. In Spain, prevalence, as determined by densitometry, is about 30% in women aged 50 years old, increasing to 50% in women older than 70 years old [6,7]. About 40% of Caucasian women will suffer at least one bone fracture after their 50s [6,7].

Strategies to minimize the prevalence of the disease and its complications would be to enhance bone mass and reduce loss at advanced ages [8]. There is convincing scientific evidence supporting the MD’s role in the reduction of overall risk for diabetes, cardiovascular, and neurodegenerative diseases. Regarding site-specific cancers and inflammatory and metabolic conditions, the evidence is suggestive or weak and is unconvincing regarding low-density lipid reduction. The MD has been shown to modestly reduce the risk of fracture and is associated with higher BMD [2]. Thus, the overall evidence points toward the role of the MD in maintaining bone health, especially in postmenopausal women [9].

Residual confounding factors in observational studies has become a major concern, with many international studies analyzing the relationship between diet and fracture risk [10]. For instance, bias should be taken into account when considering meta-analysis results on prospective cohort studies beyond the Mediterranean region [11]. This is due to the paucity of data and the lack of consensus on the definition of the MD [11]. Furthermore, physical activity has been demonstrated to be a robust indicator of a lower risk of bone fractures [10]. Since a healthier diet is related to higher physical activity levels, the latter needs also to be taken into account.

The observed inverse association between dietary quality and bone fracture risk should be interpreted with caution because the case-control design of most studies’ findings cannot indicate causality [12]. There is a lack of research into the relationship between the MD and bone fracture risk that clarifies causation or confounding factors.

A large amount of macro- and micronutrients in diet can improve bone health [5]. While Ca and vitamin D intake has conventionally been considered of importance [13], a meta-analysis of 33 randomized clinical trials (51,145 participants) concluded that Ca and vitamin D intake in the form of food supplements was not associated with a significantly reduced risk of hip fractures compared to a placebo [14]. Other essential nutrients for bone health include potassium, magnesium, and vitamin K [15,16] obtained from a wide range of foods including fruits, vegetables, and whole grains [15]. Those nutrients and many other antioxidant compounds are abundant in the MD diet, together with unsaturated fatty acids, which have a synergic effect [17]. Thus, this accumulated effect of the food components—i.e., the diet quality—is measured through dietary pattern analysis as foods are not eaten in isolation. For instance, a higher adherence to the MD is associated with higher dietary intake of polyphenols [18] and other antioxidant compounds such as vitamin C, vitamin E, carotenoids, and flavonoids [19]. MD, in turn, is poor in processed and pro-inflammatory foods, which are postulated as risk factors in some chronic diseases including osteoporosis [18]. Greater adherence to MD influences coagulation processes by lowering levels of interleukin-6, homocysteine, and fibrinogen [20]. Thus, MD alters biomarkers of inflammation and cardiovascular disease [21].

Adherence to a Westernized eating pattern (a diet high in refined cereals, processed foods, sugar and corn-syrup, saturated fat, and meat, combined with low consumption of fresh fruit and vegetables) has been linked to a greater degree of inflammation [22].

It is thought that oxidative stress and a concomitant increase in inflammation could predispose one to osteoporosis [23]. Thus, the protective effect of the MD could be attributed to its high content of antioxidants, olive oil, moderate alcohol intake, and regular physical activity.

Our hypothesis is that the MD and physical activity, as potentially modifiable risk factors affecting bone quality, could be promoted as an effective risk-reduction for osteoporotic fractures [2,9,16]. Our aim, therefore, was to study the association between the MD and bone fracture risk in a sample of middle-aged women and review the literature on the topic.

## 2. Materials and Methods

This case-control study was carried out in a primary-care health center in Girona (Spain) between March and May 2011. The sample was drawn from middle-aged women (45–65 years) with and without an OP diagnosis. The study protocol, which applied current guidelines on ethics and personal data protection, was approved by the Ethics Committee of the Girona Research Unit (IDIAP Jordi Gol) and by the management of the participating institution (Santa Clara Primary-Care Health Centre, Gerència Territorial Girona, Catalan Health Institute). A total of 290 women were selected from eCAP (a medical records database of the Autonomous Government of Catalonia): (a) 145 women with a known OP diagnosis according to World Health Organization (WHO) criteria, and (b) 145 women without an OP diagnosis as randomly selected controls. Women born between 1946 and 1966 were selected as potential perimenopausal or menopausal women, so as to avoid age as a determining factor in OP prevalence. Individuals with a diagnosis of osteopenia were ruled out as having a minimal eCAP record.

Demographic data, medical history, and lifestyle factors were assessed, using, as necessary, validated self-administered questionnaires with telephone contact included whenever necessary to clarify doubts.

### 2.1. Diet

The PREDIMED questionnaire [24] was used as a weekly food frequency questionnaire to evaluate adherence to the MD as a measure of overall dietary quality. All foods were coded as portions of normal use (e.g., a 200-mL glass, a 100-g portion, etc.) To evaluate the effect of coffee on bone mass, average daily intake was assessed on the basis of a standard 50-mL cup. Body mass index (BMI) was also recorded and Ca intake (mg/day) was measured using the INDICAT questionnaire [24,25], which also queries reasons for regularly taking Ca and vitamin D supplements and whether these were prescribed or self-administered.

### 2.2. Physical Activity

The short version of the International Physical Activity Questionnaire (IPAQ), as validated for different countries [26,27], was used to assess physical activity in the previous 7 days (on the assumption that a week would be representative of normal physical activity). The responses reflected low, moderate, or high levels of physical activity and also the activity type, activity frequency, and time dedicated to each activity.

### 2.3. Alcohol and Tobacco Consumption

Alcohol intake was measured using the validated Systematic Interview of Alcohol Consumption (ISCA), consisting of 3 questions on quantity and frequency of alcohol consumption [28,29] classified into standard units. Consumption risk in women was rated as 17 or more units/week (168 g/week). Regarding tobacco use, participants were classified as smokers, subclassified in 3 categories according to the number of cigarettes smoked/day (<10, 10–20, and >20), and non-smokers, subclassified as persons who never smoked and ex-smokers of <1 year and of >1 year.

### 2.4. Comorbidity Factors

Menstrual status, administration of hormone replacement therapy (HRT), and sun exposure levels were assessed. Individuals were classified as follows: menopausal, differentiating between women whose last period was >5 years previously, women whose last period was <5 years previously, and women whose menopause occurred before the age of 40 years (because early menopause is considered a secondary OP risk factor); perimenopausal (considering as the last menstrual cycle occurring in the previous year and the absence of regular cycles); and non-menopausal. HRT during the postmenopausal stage was classified as follows: currently administered (continuously in the previous year); previously administered (even if only for a brief period); and never administered. Finally, sun exposure was measured using a semi-quantitative scale, as active (intentional sun-seeking), regular (a very active outdoor life), occasional (a mostly indoor life), or exceptional (active avoidance of solar exposure). Finally, any drug intake or other factors that could increase OP risk were recorded.

### 2.5. Statistical Analysis

To analyze possible associations between bone fracture risk, we used student *t*-tests for the 2 groups (women with and without an OP diagnosis). Differences were analyzed for MD adherence (the MD index) and each of its components separately. Bivariate Pearson correlation coefficients were calculated to obtain linear relationships between variables. Data were further analyzed for partial correlations between variables, controlling for age and BMI. Finally, binary logistic regression models were constructed with all variables that were not highly correlated among themselves. The significance level was considered at *p* < 0.05. To obtain the final model, backward variable selection was applied, deleting from the model those that failed the Wald test. Statistical analyses were performed using SPSS v15 (SPSS Inc, Chicago, IL, USA).

## 3. Results

From the population of women born between 1946 and 1966, 145 each were selected from 5203 women with an OP diagnosis and from 5164 women without an OP diagnosis. Of the 290 women in the sample, 172 (59%) completed the survey, with the remaining 41% not replying, refusing to participate, or unlocatable. Ultimately, 139 (48%) were analyzed, 64 with an OP diagnosis, and 75 without an OP diagnosis. Noteworthy was the change in eating habits declared by 26% of the women after an OP diagnosis.

The average age was 56.78 years old and the average BMI was 24.8 (the upper limit of normal weight). Most patients (94.2%) were Spanish, and the remaining patients were South American or British. Half the patients (51.2%) reconfirmed the OP diagnosis, and 13.4% declared that they had changed their diet after disease diagnosis. Average adherence to the MD was 11.77 (maximum 12) and most patients declared using olive oil as a dressing (99.4%) and for cooking (88.4%). Average Ca intake was 855.8 mg/day, considerably lower than the recommended daily intake, and 9.3% of the women reported high coffee consumption (>4 cups/day).

Physical activity levels were mainly moderate (51.2%) followed by high (34.9%). Most women (83.1%) were non-smokers, 2.9% smoked >20 cigarettes/day, and 31.4% had stopped smoking >1 year previously. Around 2% of patients were high-risk alcohol consumers (>17 units/week), while close to two thirds (59.3%), and over a third (39%) were intermediate-and low-risk drinkers, respectively. In most cases the preferred drink was wine. In relation to menstruation, 13.4% of the women had regular cycles, 78.5% were fully menopausal and 8.1% were premenopausal. Other factors associated with OP risk were HRT (22.6%), long-term (>1 month), oral corticosteroid (CS) treatment (9.9%), diagnosis of hyperparathyroidism (2.9%), and rheumatoid arthritis (1.7%).

Since the study was case-control, diet and OP were not monitored or followed up. Instead, a filter was applied to the transversal data to identify women who did not make dietary changes after being diagnosed with OP and, in this way, to determine the relationship between diet and OP onset. Evaluated were differences between patients with OP who changed and those who did not change their diet (data not shown). While no significant changes were observed in the MD index (MDI) overall, increased consumption of certain dietary components (such as vegetables and fish) was observed. The most significant difference observed was in Ca intake. Specifically, patients declared having changed their diet increased their daily Ca intake by a median of 220 mg, mainly through the substitution of normal milk for Ca-enriched milk. Overall, the consumption of dairy products increased slightly, while normal milk consumption decreased to a statistically significant degree (data not shown).

Table 1 summarizes anthropometric data, alcohol and tobacco use, and menstrual status for women with and without OP. It can be observed that OP affects older women (average age 59.7 years) significantly more than patients without a diagnosis (54.07 years). A link can also be observed between higher BMI and OP: the non-OP group had a slightly higher BMI (25.46 kg/m^2^ vs. 24.29 kg/m^2^). Most respondents declared consuming fewer than 17 standard units of alcohol per week. Menstrual status, very linked to age, showed significant differences in relation to an OP diagnosis. Early menopause was also a risk factor, although significance was low.

Table 2 reflects differences in MD adherence, intake of specific foods, and physical activity between women with and without an OP diagnosis. There were no significant differences (*p* < 0.1) in adherence to the MD or its food components between the groups. However, Ca intake was significantly different (*p* = 0.257) at 863 mg/day for women without OP and 796 mg/day for women with OP, as was physical activity.

The correlation analysis pointed to certain variables with a strong correlation between them. Given the observed dependencies, excluded were the menstrual status, tobacco and coffee consumption, and hyperparathyroidism variables (data not shown). Since analysis revealed that age and BMI were the factors most strongly correlated with OP, a partial correlation analysis (Table 3) was conducted with the aim of determining the correlation between variables, especially with OP while controlling for age and BMI. The results point to partial correlation (coefficient −0.11 for a significance of *p* = 0.19) between OP and the MDI, despite the MDI not featuring as significant in the simple correlation.

The fracture risk assessment (FRAX) algorithm to calculate fracture risk has been analyzed as a predictor of an OP diagnosis [30]. For the 2 ways of expressing FRAX—the major osteoporotic index and the hip fracture index—we separately regressed the OP diagnosis variable, obtaining similar Nagelkerke’s R2 values in both cases (0.242 and 0.280, respectively) and a positive beta coefficient of 0.000 for significance in both cases. However, a linear regression with these indexes and the rest of the variables was ruled out, as accuracy and significance were less than in the logistic regression model that was finally selected.

Table 4 shows the influence of various variables on the OP variable for a binary logistic regression model constructed with OP diagnosis as the independent variable and MD adherence, Ca intake, IPAQ score, alcohol consumption, CS intake, HRT, sun exposure, age, BMI, and the MDI×BMI interaction variable as covariates. Older age was shown to be the most important factor in the development of OP. Other protective factors were BMI and the MD. Controlling for all other effects, including BMI, increased adherence to the MD was associated with reduced fracture risk and probability of developing OP. The positive effect of MDI×BMI interaction suggested that the MD exercised a particularly strong protective effect in patients with low BMI. The regression model yielded a negative coefficient for the alcohol variable, suggesting that low-risk consumption was not associated with an increased bone fracture risk or OP. A positive coefficient was yielded, however, for CS treatment, with the odds ratio indicating that individuals on prolonged treatment increased their bone fracture risk six-fold. Finally, Ca intake and regular physical activity also appeared as protective factors.

## 4. Discussion

Women with higher MD adherence had lower risk of bone fracture (as measured using FRAX), corroborating current scientific evidence showing an inverse relationship between bone fracture risk and the MD, which acts as a protective factor with respect to bone mass, reducing risk of fracture and maintaining a higher mean BMD [12,31]. Our results point out an especially important protective effect in patients with a low BMI. We corroborate the present scientific evidence on the Mediterranean Diet and bone health and we review these aspects in relation to lifestyle and bone health. According to Feart et al. [32], higher MD adherence was associated with a non-significant reduced risk of fractures in older individuals, while a meta-analysis by Malmir et al. [12] reported that higher adherence to the MD is associated with a 21% decreased hip fracture risk and a higher BMD in the lumbar spine, whole body, and trochanter; note, however, that these authors concluded that the likelihood of residual confounding should be considered. Higher MD adherence also tends to be associated with healthier lifestyles (greater physical activity and fewer toxic habits) [9,12]. Other studies reported 6% fewer hip fractures related to high adherence to the MD [33,34,35] and that higher MDI scores were positively and dose-dependently associated with BMD. The Rotterdam study [36] also highlighted that diets similar to the MD may be associated with lower fracture risk. In contrast, the consumption of sweets and animal products may be associated with higher fracture risk independently of BMD. The Women’s Health Initiative observational cohort study [9] reported that the high diet quality of the MD was related with low hip fracture risk, although the significance was only marginal over other diets.

In our separate analysis of the different food groups (data not shown), legumes and wine were observed to be protective factors, whereas butter and meat were risk factors. Fish and olive oil were also protective factors. Garcia-Martinez et al. [37] suggest that olive oil phenols may prevent bone mass loss by modulating the proliferative capacity and cell maturation of osteoblasts, increasing alkaline phosphatase activity, and depositing calcium ions in the extracellular matrix. In animal and human studies on bone health, Chin et al. [38] observed that olives, olive oil, and olive polyphenols act as bone protective agents, possibly by enhancing bone formation, inhibiting bone resorption and suppressing oxidative stress and inflammation. The Spanish PREDIMED trial [24] showed that the highest tertile of extra-virgin olive oil consumers had a 51% lower risk of fractures compared to the lowest tertile after adjusting for potential confounders [39]. A substudy of the PREDIMED clinical trial, PREDIMED-Reus trial, studied in 870 subjects aged 55–80 years at high cardiovascular disease risk, the relationship between dietary glycemic index, glycemic load values (DGI and DGL respectively), and the risk of osteoporotic-related fractures [40]. The study showed that higher DGI and DGL values seemed to increase the risk of osteoporotic fracture in an elderly Mediterranean population [40]. Conversely, no preventive effect on bone fracture risk was demonstrated for the consumption of vegetables, fruit, and cereals (data not shown), despite being confirmed as a positive factor in bone mineralization [15,16]. Another fundamental element in bone maintenance is Ca, mainly obtained from dairy products but also from nuts (especially almonds and walnuts), vegetables, green leafy vegetables (cabbage, spinach, etc.), and herbs. Legumes, vegetables, and fruit generally have a high Ca, iron, and phosphorus levels. According to the Dietary Reference Intakes Committee (Food and Nutrition Board, Institute of Medicine, US National Academy of Sciences, with active involvement of Health Canada), daily Ca intake differs according to age and sex. Women aged 25–50 years and postmenopausal women receiving oestrogens are recommended to consume 1000 mg/day, while postmenopausal women not receiving oestrogens and women over 65 years are recommended to consume 1500 mg/day. In our sample, average consumption was 856 mg/day, significantly less than the recommended intake. This was possibly explained by collection bias in relation to other food sources of Ca, such as water, aromatic herbs, and spices. Nonetheless, the values are similar to the Spanish mean (almost 800 mg/day) reported in a recent review of calcium intakes worldwide [41]. Moreover, 16% of the women in our study consumed functional foods that provide extra calcium intake (data not shown), with differences encountered among women with and without an OP diagnosis. We cannot conclude, however, a different use of functional or Ca-enriched foods associated with a diagnosis of OP since, in most cases, their use derives from a medical prescription motivated by the diagnosis.

Regarding risk factors, alcohol abuse is known to have negative effects on bone formation, as it is associated with the onset of metabolic bone disease and the appearance of fractures [7,28]. However, previous studies have suggested that drinking alcohol in small quantities does not increase OP risk [28,29]; this is especially relevant to post-menopausal women because alcohol can modify androgen and oestrogen concentrations [28,29]. Nonetheless, the harmful effects of alcohol consumption on health cannot be overlooked.

Another risk factor is coffee intake, which has not been considered a significant risk in other studies when accompanied by appropriate Ca consumption [7,42]. In our study, most women consumed a moderate amount of coffee (<4 cups a day), and we found no association with bone fracture risk.

Several epidemiological studies have found a link between smoking and reduced BMD values and an increased incidence of vertebral and hip fractures. The detrimental effects of smoking may be due to decreased osteoblastic activity, interference with intestinal Ca absorption and its anti-oestrogen effect. Some studies reject these harmful effects, attributing the negative relationship between smoking and bone metabolism disorders in women to lifestyle differences between smokers and non-smokers. We found no significant relationship but did find that bone fracture risk increased proportionally to the number of cigarettes smoked, as reported elsewhere [28]. Furthermore, smokers typically consumed more alcohol and coffee, resulting in a synergic effect.

Physical activity plays an important protective role in OP during adolescence by enhancing bone mineral density and at more advanced ages by repairing and maintaining muscle pressure and tension. Increased physical activity is also associated with a higher intake of protein, fiber, iron, magnesium, zinc, potassium, and vitamins [7]. The level of physical activity observed in our study was high (86% reported moderate or high physical activity levels), and we could confirm a significant preventive effect on bone fracture risk. Therefore, it is important to promote physical activity, most especially among women at high risk, e.g., exercise based on building muscular strength (2 days per week) and balance (3 days per week) [43] to strengthen muscles and improve coordination [44]. An evidence-based analysis [45] concludes that long-term physical activity reduces falls significantly, e.g., tai chi [46,47,48]. The prescription of physical activity and exercise as a strategy to treat or prevent OP needs to be individualized, however, according to each patient’s characteristics and conditions. According to different studies in support of current guidelines [49,50,51], goals for OP should include exercises for pain reduction, enhanced mobility, and improvements in muscle endurance, balance, and stability [52,53].

An independent variable for OP is age, related to other factors associated with the disease, such as less osteoblastic activity, changes in body composition, reduced intestinal absorption of Ca, nutritional deficiencies (especially of vitamin D), low sun exposure, and sedentary behavior [24]. In our sample, 78% of women were menopausal and 8.1% had early menopause (either spontaneous or surgical) and, linked as it is to oestrogen levels, menopause is the determining factor in the pathogenesis of OP. In addition to mechanical physical factors, humoral factors such as hormonal interrelations, calcitonin, parathormone, and vitamin D3 regulate the bone remodeling process, affecting the risk of developing the disease [54]. The risk is greatest at earlier ages and when hormonal deprivation is abrupt (e.g., due to surgical menopause).

Regarding BMI, the MD seems to have a protective effect on fracture risk for patients with a low BMI [32]. Indeed, both weight gain and weight loss have both been associated with increased bone fracture incidence [55]. A BMI <19, indicating a lack of fat, is associated with significantly lower BMD, which seems to be related to a lower osteoblastic effect and a lower brake on osteoclastic activity resulting from reduced oestrogen production [56,57,58]. While we found a significant relationship between BMI and bone fracture risk, this could not be associated with a higher or lower proportion of women with a BMI at risk (<19).

It has been observed that 30% of women with early menopause go undiagnosed with OP. Such patients need to be monitored by clinical evaluation of their risk of fracture and be encouraged to follow a proper diet, ensure adequate Ca intake, and avoid modifiable risk factors. The scientific recommendations point to a prevention strategy to avoid falls and the use of drugs such as bisphosphonates whenever possible.

Interestingly, there is no consensus on the definition of OP, which makes it is difficult to use comparable tools for its diagnosis. We used the FRAX to evaluate fracture risk based on risk factors and BMD measurement at the femoral neck. Although FRAX has been extensively used in the UK [59], it is not widely used in Spain. The FRAX-Spain risk scale underestimates the risk of major fracture in some cohorts, with the risk higher in women at low risk, lower in women >65 years, and in women with densitometry-confirmed OP or with a previous fracture history [60]. Johansson et al. [61] concluded in 2017 that targeting fracture probabilities, rather than BMD alone, identifies women at higher fracture risk.

Our study has several limitations. First, the small sample (*n* < 300) may not be representative of the general population and information about existing endocrine diseases, educational, and socio-economic status of the sample is lacking, which could have potentially influenced the findings. Second, the self-administered food intake questionnaire may imply a nutritional data collection bias. More exhaustive and reliable food intake data would require assessment over a week. Note that an a priori dietary index to assess the cumulative effects of different foods on health would be a useful tool to investigate diet quality in epidemiological studies [7,57]. Third, the MDI used in this study to predict fracture incidence and bone health was not initially designed for that purpose. Fourth, the date of OP diagnosis was not recorded. Fifth, the FRAX method has several limitations, especially in terms of clinical risk factors (CRF) identifying medium to high risk cases (≥2 high CRF, or ≥4 moderate CRF, or ≥1 high CRF + ≥2 moderate CRF) [62]. Sixth, while we recorded drugs that possibly increased the likelihood of OP, we did not record the use of anti-osteoporotic drugs such as bisphosphonates and denosumab. Finally, further studies should record breast cancer histories and subsequent treatments that can cause menopause and influence bone health.

Regarding pharmacological treatment to prevent OP, some advances have been made with glucocorticoid-induced OP in postmenopausal women and men at a high risk of fragility fracture. In regard to supplements, the evidence is contradictory. Some studies point to the benefit of vitamin D supplements in people at a major risk of fall, e.g., 12 months of 800 IU/day [43] or 700 IU/ day of vitamin D [44], while the American Association of Clinical Endocrinologists’ guidelines [63] report that vitamin D improves muscular strength. There is also evidence in support of vitamin D co-administered with Ca reducing fracture risk [64]. However, several reviews [62,65,66,67,68] state that there is limited evidence to support supplementation of vitamin D, as benefits in relation to the risk of falls have not been demonstrated for the general population except when there is a deficit of vitamin D. A recent study by Bolland et al. [69] suggests that vitamin D supplementation does not prevent fractures or falls nor does it have clinically meaningful effects on BMD. In all, there seems to be little justification to use vitamin D supplements to maintain or improve musculoskeletal health [69]. In fact, the American College of Physicians [70] has revised its statement on vitamin D efficacy regarding the risk of fractures. Conflicting evidence has also been reported for Ca supplements in reducing fractures in older men and women (level 2 or mid-level evidence), although the evidence suggests that it may reduce BMD loss in postmenopausal women and total body mass loss in women over 65 years old. Vitamin D supplementation alone does not reduce fracture risk (level 2 or mid-level evidence), while calcium plus vitamin D supplementation appears to reduce fractures in older adults (particularly in institutionalized settings) but not in community-dwelling individuals (level 2 or mid-level evidence). The American Geriatrics Society consensus recommends taking ≥1000 units/day (25 mcg/day) of vitamin D plus 1000–1200 mg/day of Ca to reduce risk of fracture and falls, but those values cannot be extrapolated to the general population. Overall, therefore, there is no conclusive evidence of the benefits of Ca or vitamin D supplements alone or together in terms of preventing fractures, falls and BMD loss in older men or women or in postmenopausal women [70].

## 5. Conclusions

Our findings support the idea that a healthy lifestyle should be the first option to avoid bone fractures—e.g., following the MD as a non-pharmacological preventive strategy, which includes regular physical activity, especially outdoors, and eliminating or reducing toxic habits, such as smoking and excess alcohol and caffeine intake.

Further studies with larger samples and longitudinal data are needed to investigate the factors that influence diet in maintaining bone mass and bone health. Scientific evidence is needed in order to properly educate patients and professionals in changing lifestyle habits and transmit clear messages. Our results corroborate strong evidence based on cohort, control, and trial studies of intermediate risk factors in support of the MD to prevent certain chronic diseases. We found the MD to be associated with higher BMD and lower fracture risk, probably due to the combined synergistic effect of its components; therefore, according to current scientific evidence, adherence to the MD is a protective factor in the maintenance of bone mass and the prevention of bone fracture risk. The inclusion of a Mediterranean-style nutritional approach in clinical practice could potentially improve the well-being of patients.

## Figures and Tables

**Table 1:** Descriptive data on the cohort of women indicating the population means of anthropometric, alcohol and tobacco consumption, and menstrual status in participating women by osteoporosis status.

	Osteoporosis		T	*p*	95% CI ^1^	
	Yes (*n* = 64)	No (*n* = 75)			Lower	Upper
Age (years)	59.7 ± 4.4	54.07 ± 5.6	6.50	<0.001 *	3.92	7.35
BMI (kg/m^2^)	24.29 ± 3.7	25.46 ± 4.4	−1.68	0.096	−2.55	0.21
Tobacco ^2^	0.13 ± 0.3	0.24 ± 0.4	−1.74	0.084	−0.25	0.02
Alcohol ^3^	0.53 ± 0.5	0.67 ± 0.5	−1.63	0.105	−0.30	0.03
Menstrual status ^4^						
Regular cycles	0.02 ± 0.1	0.25 ± 0.4	−4.20	<0.001 *	−0.35	−0.13
Perimenopause	0.02 ± 0.1	0.13 ± 0.3	−2.61	0.010 *	−0.21	−0.03
Menopause	0.97 ± 0.2	0.61 ± 0.5	5.50	<0.001 *	0.23	0.48
Early menopause	0.13 ± 0.3	0.05 ± 0.2	1.50	0.136	−0.02	0.17

^1^ Confidence interval of 95%. ^2^ Smokers were coded 1 and non-smokers were coded 0. ^3^ Alcohol consumption was coded 1 for moderate or high risk and 0 for low risk. ^4^ Menstrual status was coded 1 if menstruating and 0 for full menopause. * Indicates statistical significance (*p*-value < 0.05). Abbreviations: BMI: body mass index; T: T statistic.

**Table 2:** Mediterranean diet adherence, intake of specific foods (portions/day) and physical activity data in participating women by osteoporosis status (given in mean values).

	Osteoporosis		T	*p*	95% CI ^1^	
	Yes (*n* = 64)	No (*n* = 75)			Lower	Upper
Physical activity ^2^	0.81 ± 0.4	0.89 ± 0.3	−1.35	0.178	−0.20	0.04
MDI	11.81 ± 1.8	11.96 ± 1.8	−0.49	0.626	−0.74	0.45
Olive oil	2.88 ± 1.6	3.05 ± 1.7	−0.64	0.524	−0.73	0.37
Vegetables	2.13 ± 1	2.07 ± 1.1	0.28	0.782	−0.32	0.42
Fruit	2.67 ± 1.4	2.41 ± 1.3	1.15	0.253	−0.19	0.72
Meat	2.81 ± 2.1	2.65 ± 2.4	0.42	0.676	−0.59	0.91
Butter	0.6 ± 1.5	0.38 ± 1	1.01	0.315	−0.21	0.66
Soda	0.87 ± 2.2	1.04 ± 2.2	−0.47	0.640	−0.90	0.56
Wine	1.66 ± 2.9	2.32 ± 3.7	−1.16	0.250	−1.80	0.47
Legumes	2.1 ± 1.2	2.15 ± 1	−0.25	0.803	−0.40	0.31
Fish	2.68 ± 1.6	2.72 ± 1.5	−0.15	0.878	−0.56	0.48
Bakery products	1.66 ± 2.6	1.71 ± 2.2	−0.12	0.903	−0.85	0.75
Nuts/seeds	2.96 ± 3.1	2.81 ± 2.7	0.31	0.755	−0.82	1.13
Ca (mg/day)	796.00 ± 351.1	863.63 ± 347.6	−1.14	0.257	−185.14	49.90
Dairy products	13.73 ± 8.9	13.64 ± 7.7	0.06	0.951	−2.69	2.87
Milk	3.95 ± 4.3	4.24 ± 4.3	−0.40	0.687	−1.74	1.15
Ca-enriched milk	0.89 ± 3.2	0.68 ± 2	0.47	0.636	−0.67	1.09
Coffee ^3^	0.02 ± 0.1	0.15 ± 0.4	−2.80	0.006 *	−0.22	−0.04

^1^ Confidence interval of 95%. ^2^ Physical activity coded 1 for moderate or high activity and 0 for low activity. ^3^ Coffee consumption was coded 1 for >4 cups/day and 0 otherwise. * Indicates statistical significance (*p*-value < 0.05). Abbreviations: Ca, calcium; MDI, Mediterranean diet index; Ca: calcium index; T: T statistic.

**Table 3:** Correlations of variables of interest, adjusted by age and body mass index.

		MDI	Ca	IPAQ	Alcohol	CS	Hormones	Sun	OP
MDI	Corr ^1^	1.00	0.05	0.08	0.09	−0.06	−0.09	0.10	−0.11
	*p* ^2^		0.55	0.35	0.32	0.46	0.30	0.22	0.19
Ca	Corr ^1^	0.05	1.00	0.03	−0.08	0.04	0.03	0.03	−0.08
	*p* ^2^	0.55		0.69	0.33	0.63	0.72	0.73	0.33
IPAQ	Corr ^1^	0.08	0.03	1.00	−0.10	−0.14	0.09	−0.04	−0.08
	*p* ^2^	0.35	0.69		0.22	0.11	0.27	0.64	0.33
Alcohol	Corr ^1^	0.09	−0.08	−0.10	1.00	0.04	−0.02	0.02	−0.16
	*p* ^2^	0.32	0.33	0.22		0.65	0.83	0.81	0.06
CS	Corr ^1^	−0.06	0.04	−0.14	0.04	1.00	0.00	0.00	0.13
	*p* ^2^	0.46	0.63	0.11	0.65		0.98	0.99	0.14
Hormones	Corr ^1^	−0.09	0.03	0.09	−0.02	0.00	1.00	0.00	0.05
	*p* ^2^	0.30	0.72	0.27	0.83	0.98		0.98	0.56
Sun	Corr ^1^	0.10	0.03	−0.04	0.02	0.00	0.00	1.00	0.07
	*p* ^2^	0.22	0.73	0.64	0.81	0.99	0.98		0.40
OP	Corr ^1^	−0.11	−0.08	−0.08	−0.16	0.13	0.05	0.07	1.00
	*p* ^2^	0.19	0.33	0.33	0.06	0.14	0.56	0.40	

^1^ Pearson’s correlation. ^2^ Two-tailed significance. Abbreviations: Ca, calcium; CS, corticosteroids; IPAQ, International Physical Activity Questionnaire; MDI, Mediterranean diet index; OP, osteoporosis; Sun, sun exposure.

**Table 4:** Results of the adjusted binary logistic regression model.

	B	Wald	*p*	95% CI for EXP (B)	
				Lower	Upper
MDI	−2.137	5.979	0.014 *	0.021	0.654
Ca	−0.001	2.196	0.138	0.998	1.000
IPAQ	−0.878	1.795	0.180	0.115	1.502
Alcohol	−1.127	5.417	0.020 *	0.125	0.837
CS	1.830	4.972	0.026 *	1.248	31.153
Age	0.255	28.614	<0.001 *	1.175	1.417
BMI	−1.189	7.455	0.006 *	0.130	0.715
MDIxBMI	0.084	5.623	0.018 *	1.015	1.167
Constant	17.499	2.757	0.097		

Abbreviations: BMI, body mass index; Ca, calcium intake; CI, confidence interval; CS, corticosteroids; IPAQ, International Physical Activity Questionnaire; MDI, Mediterranean diet index; OP, osteoporosis. * Indicates statistical significance (*p*-value < 0.05).
